# Habitat Loss other than Fragmentation per se Decreased Nuclear and Chloroplast Genetic Diversity in a Monoecious Tree

**DOI:** 10.1371/journal.pone.0039146

**Published:** 2012-06-18

**Authors:** Xin Zhang, Miao-Miao Shi, Dong-Wei Shen, Xiao-Yong Chen

**Affiliations:** 1 School of Resources and Environmental Sciences, Tiantong National Observation Station for Forest Ecosystems, East China Normal University, Shanghai, China; 2 South China Botanical Garden, China Academy of Sciences, Guangzhou, China; 3 Department of Community Ecology, Helmholtz Centre for Environmental Research–UFZ, Halle, Germany; CNR, Italy

## Abstract

Generally, effect of fragmentation per se on biodiversity has not been separated from the effect of habitat loss. In this paper, using nDNA and cpDNA SSRs, we studied genetic diversity of *Castanopsis sclerophylla* (Lindl. & Paxton) Schotty populations and decoupled the effects of habitat loss and fragmentation per se. We selected seven nuclear and six cpDNA microsatellite loci and genotyped 460 individuals from mainland and island populations, which were located in the impoundment created in 1959. Number of alleles per locus of populations in larger habitats was significantly higher than that in smaller habitats. There was a significant relationship between the number of alleles per locus and habitat size. Based on this relationship, the predicted genetic diversity of an imaginary population of size equaling the total area of the islands was lower than that of the global population on the islands. Re-sampling demonstrated that low genetic diversity of populations in small habitats was caused by unevenness in sample size. Fisher's *α* index was similar among habitat types. These results indicate that the decreased nuclear and chloroplast genetic diversity of populations in smaller habitats was mainly caused by habitat loss. For nuclear and chloroplast microsatellite loci, values of F_ST_ were 0.066 and 0.893, respectively, and the calculated pollen/seed dispersal ratio was 162.2. When separated into pre-and post-fragmentation cohorts, pollen/seed ratios were 121.2 and 189.5, respectively. Our results suggest that habitat loss explains the early decrease in genetic diversity, while fragmentation per se may play a major role in inbreeding and differentiation among fragmented populations and later loss of genetic diversity.

## Introduction

Habitat fragmentation is a landscape-scale process involving both habitat loss and the breaking apart of habitats, i.e. narrow sense fragmentation or fragmentation per se [1]. Habitat loss leads directly to the loss of individuals or entire populations, and therefore results in loss of genetic diversity; decreased population sizes in the remnant habitats and increased isolation will alter the genetic composition of a population through genetic drift and inbreeding [2]. These genetic changes may in turn leave the species more vulnerable to demographic and environmental stochasticity and lead to negative impacts on the persistence of remnant populations [2]–[4]. Although the importance of habitat loss and fragmentation per se in genetic variation has been recognized, their relative importance has not yet been well studied.

A commonly adopted method of identifying the genetic consequences of habitat fragmentation is to study the relationship between genetic diversity and habitat size or population size (for example, Zhao et al. [5]). A positive relationship between them was regarded as evidence of the negative consequences of habitat fragmentation, which has been frequently observed in empirical studies [2]–[4]. However, such observations mixed the effects of habitat loss and fragmentation per se [1]. Both habitat loss and fragmentation per se can lead to such a positive relationship. Therefore, we should decouple the distinct roles of habitat loss and fragmentation per se in the loss of genetic diversity.

Contrary to habitat islands, real islands have clear boundaries, which make it relatively easy to obtain the landscape parameters, such as area, distance to adjacent habitats, etc [6]. Islands have long been recognized as natural laboratories of evolution and many studies have provided evidence supporting theoretical predictions [7], [8]. However, few studies have focused on recently formed land-bridge islands [9], and tracked changes in genetic composition of island populations. Damming rivers to develop hydroelectric stations provides an opportunity to conduct such studies [10]. Qiandao Lake, impounded in 1959, covers an area of 573 km^2^ and created more than 1000 islands of different sizes. Abundant islands, differing in size and experiencing simultaneous fragmentation, provide a special fragmentation system to study consequences of habitat fragmentation, including in genetic composition of plant populations.

In this study, we examined genetic diversity of *Castanopsis sclerophylla* (Fagaceae) populations in island habitats of different sizes and adjacent mainland habitats in the Qiandao Lake region. The focal species *C. sclerophylla* is a dominant species of evergreen broad-leaved forests, and is the most common evergreen broadleaved tree in the Qiandao Lake region [11]. The monoecious *C. sclerophylla* is wind-pollinated and gravity-dispersed, but rodents may play a role in the secondary dispersal of seeds. For such a species, formation of land-bridge islands may have a minor impact on the extensive pollen flow, but the water surrounding the islands is a critical barrier for seed dispersal. Available studies show that, in general, seed dispersal is much more limited than pollen dispersal [12], and therefore, might be more sensitive to habitat fragmentation. In fact, strengthened fine-scale spatial genetic structure was observed in a moderately fragmented habitat with the presence of extensive pollen flow [13]. However, most empirical studies are exclusively focused on nuclear genetic diversity, which is contributed by both parents and mediated by both pollen and seed dispersals. Responses of seed-mediated cpDNA or mtDNA genetic diversity to habitat loss and fragmentation per se are less well understood [14]–[16]. In species of Fagaceae, nuclear markers are biparentally inherited and transmitted through both seed and pollen, while chloroplast DNA is maternally inherited and transmitted through seed only [17].

In the present study, we used polymorphic microsatellite loci of both nuclear and chloroplast genomes to detect genetic composition of *C. sclerophylla* populations on islands varying in size and on adjacent mainland. Because losses of individuals and associated genetic diversity happen simultaneously with the loss of habitats that they occur, whereas genetic diversity of fragmented populations is lost slowly over subsequent generations [18], we hypothesize that at early stage of fragmentation, habitat loss plays a more important role in genetic diversity loss than fragmentation per se. Specifically we aimed: (1) to find whether nuclear genetic variation differentiated significantly among populations in habitats of different sizes, (2) to decouple the impacts of habitat loss from that of fragmentation per se on genetic diversity, and (3) to reveal whether cpDNA genetic diversity responds to habitat loss and fragmentation per se in a manner similar to nuclear genetic diversity.

## Materials and Methods

### Ethics statement

The study involved a common tree species, which is not endangered or protected species. The study location Qiandao Lake region is neither privately-owned nor protected area. Therefore, no specific permissions were required for the study.

### Study sites

**Figure 1:**
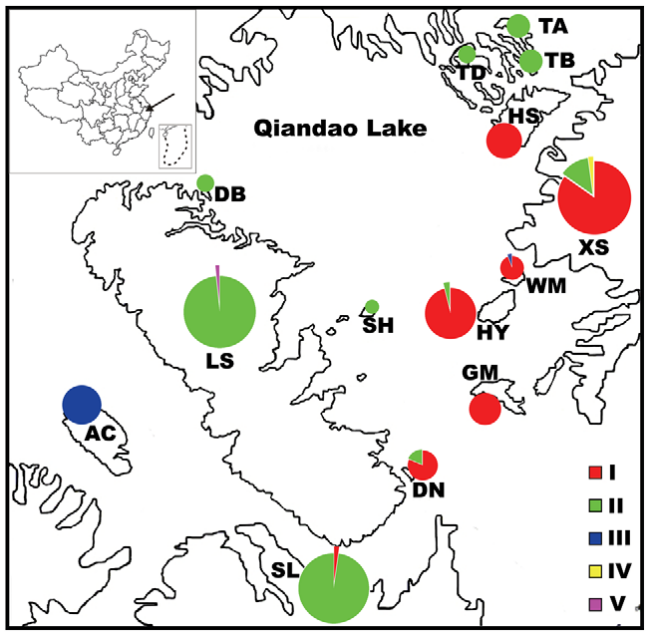
Sampling sites and distribution of chloroplast DNA haplotypes of *Castanopsis sclerophylla* populations on islands and adjacent mainland. Pie sizes are proportional to sample size. Population codes are the same as in table 1.

The study sites were located in Qiandao Lake National Forest Park (QLFP) in Zhejiang Province, China (Fig. 1). The mean annual precipitation in this region is 1381.5 mm, and the mean annual temperature is 17.0°C, with an average temperature of 28.9°C in July and 5.0°C in January [19]. The potential zonal vegetation of the study area are evergreen broadleaved forests (EBLFs) dominated by *Castanopsis sclerophylla* and *Cyclobalnopsis glauca* (Thunb.) Oersted ( = *Quercus glauca* Thunb) [20]. However, most evergreen broadleaved forests at low elevations were harvested during construction of the dam. The current vegetation is dominated by the wind-dispersed pioneer tree, *Pinus massoniana* Lamb. Pure *P. massoniana* forests are estimated to dominate 60% of the land cover in this region [19].

There are 1078 islands larger than 2500 m^2^ when the water-level is 108.0 m a.s.l., the altitude of the dam, and several hundred of smaller islands in this impoundment. Previous surveys indicated *C. sclerophylla* occurred mainly on islands and adjacent mainland in the southeastern sub-region of QLFP [11]. This sub-region includes several dozen islands, and *C. sclerophylla* was found on 13 islands, including Laoshan Island (LS, 874 hm^2^), the largest one. The peak altitudes of the islands range from 105.4 m to 408.5 m. Leaves of *C. sclerophylla* were collected from 11 of the 13 island populations, except two extremely small populations of less than 5 individuals, and 3 mainland populations in QLFP (Fig. 1). Table 1 shows basic information about the islands. We classified the sites into three habitat types: 1) slightly fragmented habitats (SL, XS, and TA) on the mainland, 2) moderately fragmented habitats, including islands>10 hm^2^ (islands LS, AC, HS, HY, and GM), and 3) strongly fragmented habitats, including islands <10 hm^2^ (islands TB, TD, WM, SH, DN and BN) (Table 1, Fig. 1).

**Table 1:** Basic information of *Castanopsis sclerophylla* populations and the parameters of genetic diversity in the present study.

Populations	Code	A (hm^2^)	D (km)	*N*	*N_S_*	*N*_A_	*A*_R_	*H*_O_	*H*_E_	*H*_cpDNA_
Slightly fragmented										
Shilin	SL	885.5	-	>4000	46(27, 19)	4.57(4.14, 4.29)	3.08(2.98, 3.12)	0.360(0.407, 0.293)	0.509(0.507, 0.512)	0.007
Xianshan	XS	2567.0	-	>4000	46(26, 20)	4.71(3.86, 4.14)	3.20(3.00, 3.17)	0.348(0.352, 0.343)	0.521(0.503, 0.526)	0.045
Taiping	TA	405.0	-	>4000	26(19, 7)	4.00(3.86, 3.43)	2.98(2.92, 3.17)	0.371(0.351, 0.425)	0.496(0.480, 0.508)	0
Moderately fragmented										
Laoshan	LS	874.91	0.09	>4000	58(30, 28)	3.71(3.57, 3.43)	2.82(2.69, 2.78)	0.271(0.257, 0.291)	0.461(0.421, 0.472)	0.006
A-ci	AC	50.58	0.39	400	59(32, 27)	4.43(4.29, 4.14)	3.15(3.09, 3.01)	0.304(0.299, 0.310)	0.515(0.504, 0.495)	0
Huangshan	HS	39.80	0.27	500	47(30, 17)	4.29(4.14, 3.43)	3.20(3.23, 2.83)	0.273(0.280, 0.261)	0.538(0.542, 0.504)	0
Guanmiao	GM	15.60	0.72	100	31(25, 6)	3.57(3.43, 2.57)	2.57(2.49, 2.47)	0.387(0.343, 0.571)	0.431(0.422, 0.446)	0
Heyang	HY	13.00	0.15	350	50(32, 18)	4.43(4.00, 3.86)	2.96(2.89, 3.06)	0.289(0.304, 0.262)	0.508(0.498, 0.522)	0.013
Strongly fragmented										
Taiping island B	TB	5.72	0.07	50	20(14, 6)	3.57(3.57, 2.57)	2.81(2.91, 2.49)	0.293(0.327, 0.214)	0.452(0.460, 0.409)	0
Taiping island D	TD	3.90	0.15	40	9(8, 1)	3.00(2.57, 1.43)	2.74(2.49, NA)	0.302(0.286, 0.429)	0.500(0.476, 0.429)	0
Wuming	WM	3.18	0.15	21	17(17, 0)	3.43(3.43, NA)	2.72(2.72, NA)	0.261(0.261, NA)	0.426(0.426, NA)	0.019
Shihu	SH	1.50	0.58	6	5(5, 0)	3.00(3.00, NA)	3.00(3.00, NA)	0.314(0.314, NA)	0.460(0.460, NA)	0
Dongnan	DN	1.12	0.09	32	32(26, 6)	3.71(3.57, 3.00)	2.91(2.88, 2.83)	0.371(0.374, 0.357)	0.496(0.498, 0.483)	0.052
Dongbei	DB	0.63	0.15	14	14(13, 1)	3.57(3.57, 1.29)	3.05(3.06, NA)	0.276(0.275, 0.286)	0.481(0.488, 0.286)	0

A: habitat size (hm^2^); D: distance to the nearest landmass (km); *N*: estimated or real population sizes; *NS*: sample size; *N*_A_: number of alleles per locus; *A*_R_: allelic richness, based on minimal sample size of 5 individuals, post-fragmentation cohorts with only 1 individual were excluded for calculation; *H*_O_: observed heterozygosity; *H*_E_: expected heterozygosity; *H*_cpDNA_: Nei's gene diversity based on cpDNA microsatellite data. Values of pre-and post-fragmentation cohorts are shown in parenthesis.

NA: not available.

### Sample collection

Estimated or true population sizes of the sampled populations ranged from 6 to more than 4000 (Table 1). In large and medium-sized populations (>40 individuals), fresh leaf samples were collected randomly. A distance of at least 20 m was maintained between samples. In small populations, all the individuals were sampled. To infer the effect of the fragmentation process on *C. sclerophylla* populations, the samples were classified into two cohorts: pre-fragmentation and post-fragmentation, based on the age of the individuals. The classification was based on the relationship between basal diameter and age constructed by Zhang [21] in the same sub-region during the surveys. Healthy fresh leaves from each individual were collected and dried with silica gel in a sealable bag.

### DNA extraction and genotyping

Total DNA was extracted with a modified CTAB procedure [22], from approximately 0.05 g of dry leaf material which had been ground to a fine powder using a Fastprep. The genotype of each individual was determined at seven polymorphic, nuclear microsatellite loci (Ccu16H15, Ccu33H25, Ccu62F15, Ccu87F23, Ccu90T17, Ccu93H17 and Ccu97H18) developed for *C. cuspidata* var. *sieboldii* [23], [24]. The PCR reactions were performed in a DNA Engine DYAD^TM^ thermocycler (MJ Research Inc, Watertown, Mass.). A denaturation period of 4 min at 94°C was followed by 36 cycles of 40 s at 94°C, 30 s at 55°C and 40 s at 72°C, and then 10 min at 72°C for final extension. The PCR products were separated in 6% polyacrylamide denaturing gels. After electrophoresis, bands were revealed using the following modified silver-staining procedure.

Primers for chloroplast microsatellite loci developed for species of the Fagaceae [25], [26] were screened from samples of *C. sclerophylla*. Six primer pairs produced clear and distinguishable bands, and among them three were polymorphic. The six pairs of primers were applied to all samples.

### Analyses of genetic structure

The nuclear microsatellite data were used to test for linkage disequilibrium for each locus pair across all populations in FSTAT version 2.9.3.2 [27]. FSTAT was also used to calculate the number of alleles per locus (*N*_A_) and allelic richness (*A*_R_) [28]. The observed (*H*_O_) and expected (*H*_E_) heterozygosities were calculated using TFPGA [29]. The inbreeding coefficient (*F*_IS_) was estimated as the average for each population, and also for pre-and post-fragmented cohorts using FSTAT [27]. ANOVA was used to test the significance of differences in genetic variation among habitat types, and post hoc Tukey HSD was used to check the source of difference with package ′*stats*′ in R environment [30]. Relationships between habitat size and parameters of genetic diversity were analyzed in R environment.

Genetic differentiation among populations was estimated for both nuclear DNA and cpDNA data set (θc) using TFPGA [29]. The distribution of the 95% confidence intervals for *F*_ST_ was estimated using bootstrap analysis in TFPGA using loci as replicates to determine if *F*_ST_ estimates were significantly different from a null hypothesis of panmixia [29].

When a population has gone through a severe and recent genetic bottleneck, it will show a transient excess of heterozygotes [31]. A sign test [32] was performed using the program BOTTLENECK to determine if fragmentation has caused a genetic bottleneck in *C. sclerophylla* populations [33], and all loci were assumed to fit the step mutation model (SMM) or two-phase model (TPM).

The pollen-to-seed dispersal ratio (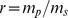) was estimated using the method of Ennos [34]. Under the assumption of an island model of dispersal, at migration-drift equilibrium, the ratio (*r*) of the amount of dispersal by pollen (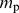) to the amount of dispersal by seed (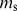) can be inferred from F-statistics estimated for biparental markers (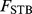) and for uniparentally inherited maternal markers (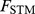).

Separation of the relative effects of habitat loss and fragmentation per se on genetic diversity

[We used three analytical methods to infer the relative roles of habitat loss and fragmentation per se in the loss of genetic diversity.

We assessed the relationship between sample size and number of alleles detected in pre-or post-fragmentation cohorts of each habitat type to account for potential biases resulting from unequal sample sizes. We checked allele accumulation curves or the number of alleles for a certain number of sampled individuals in pre-or post-fragmentation cohorts of each habitat type using SPECACCUM using the software package ‘*vegan*’ written by Oksanen et al. [35]. We used the “random” method with 10000 permutations to obtain the mean accumulation curves and their standard deviations. We also calculated Fisher's α, of pre-or post-fragmentation cohorts of each habitat type using the FISHER.ALPHA in ‘*vegan*’ [35]. Fisher's α is a satisfactory scale-independent indicator of diversity, and is defined by the following formula [36]: *S* = αln(1+n/α), where *S* is number of alleles, n is number of individuals and α is the Fisher's α.

We followed the general logic of Yaacobi *et al*. [37] to obtain an extrapolation of the genetic diversity-area relationship. We established the relationship between parameters of genetic diversity (y) and habitat area (x) using regression analyses with R version 2.14.2 [38]. If there was a significant relationship, we substituted the cumulative area of the island habitats into the regression equation and solved for the expected genetic diversity in a hypothetical habitat of the same cumulative area [39]. The value of the genetic diversity at that point represents the predicted value of an imaginary population with that particular habitat size, providing fragmentation per se has no effect on genetic diversity. If the actual genetic diversity in the set of the fragmented populations is less than this predicted value, fragmentation per se will have depressed genetic variation. If it is greater, fragmentation per se will have increased genetic diversity [37].

We also examined the relative roles of habitat loss and fragmentation per se on genetic diversity using Quinn and Harrison's [40] approach. We plotted the cumulative number of alleles-area curves in two ways: 1) starting with the smallest island and successively adding larger islands, and 2) starting with the largest island and successively adding smaller islands. Under a null hypothesis that the spatial structure of the habitat does not affect number of alleles, the two curves will be identical. If the small-islands-first cumulative allele-area saturates more rapidly than the large islands-first curve, then fragmentation per se has positive effects on number of alleles, and *vice versa*.

## Results

### Genetic diversity at nuclear microsatellite loci

Across the 460 individuals scored, seven polymorphic loci produced a total of 39 alleles. The number of alleles per locus ranged from 2 (Ccu90) to 10 (Ccu93), and H_E_ ranged from 0.013 (Ccu90) to 0.823 (Ccu33). At the population level, mean number of alleles per locus ranged from 3.00 in strongly fragmented populations SH and TD to 4.71 in the mainland population XS. There was a significant difference in mean number of alleles per locus (*N*_A_) among different habitat types (one-way ANOVA, F_2,13_ = 9.9356, P = 0.003). The post hoc Tukey HSD test indicated values of *N*_A_ in slight-and moderate-fragmented habitats were significantly higher than that of strongly-fragmented habitats. There was no significant difference in *H*_O_ and *H*_E_ among habitat types.

Using the program BOTTLENECK, no population was found to have experienced a recent bottleneck under the models of TPM or SMM. No significant relationship was found between distance to the nearest occupied habitat and parameters of genetic diversity of islands.

**Figure 2:**
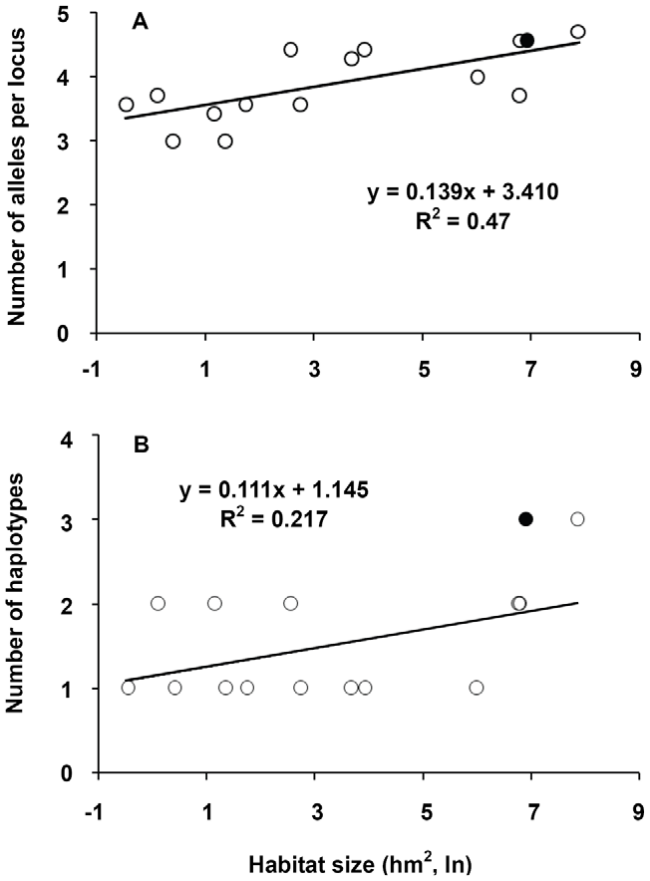
Relationships between habitat size and allele number (A) or haplotype number (B) of *Castanopsis sclerophylla* populations in the different island habitats (open circles) and their total numbers of alleles or haplotypes (filled circles). The line shows the expected number of alleles or haplotypes if fragmentation per se has no effect.

**Figure 3:**
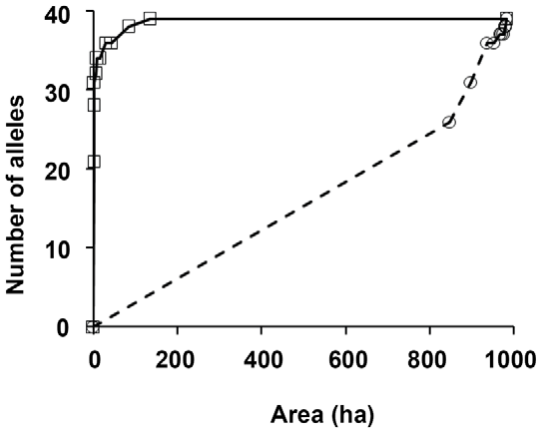
Cumulative allele-area curves for *Castanopsis sclerophylla* in Qiandao Lake region. Solid line: islands ranked smallest to largest; dashed line: islands ranked largest to smallest.

The number of alleles per locus (y) was significantly related to habitat size (*x*), and the following relationship was determined: *y* = 3.410+0.139 ln(*x*) (Fig. 2) (adjusted *R* ^2^ = 0.4258, P = 0.007). Using this equation, we may obtain the predicted value for the number of alleles per locus of an imaginary population of size equaling the total area of the islands. The predicted value (4.37) is smaller than the value of the global population of all the islands (4.57) (Fig. 2), indicating that fragmented habitats might have maintained more genetic diversity than a large one. The small-islands-first cumulative allele richness-area saturates more rapidly than the large islands-first curve (Fig. 3), also indicating fragmentation per se had positive effects on allele richness. There were no significant relationship between habitat size and expected or observed heterozygosities.

**Figure 4:**
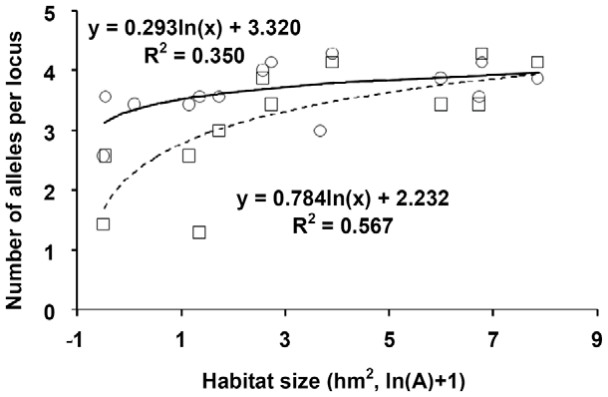
Relationships between habitat size and number of alleles per locus in pre-(circles, solid line) and post-fragmentation (squares, dashed line) cohorts of *Castanopsis sclerophylla*.

**Figure 5:**
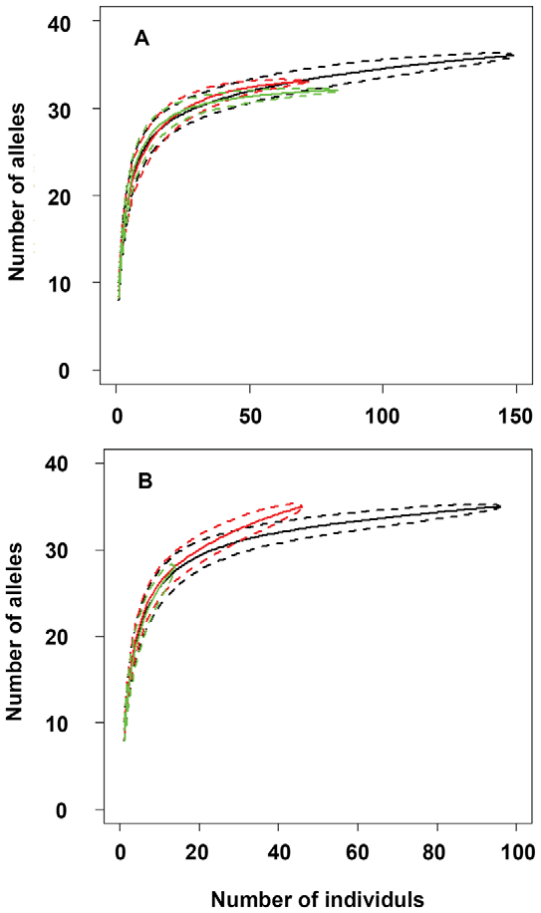
The number of alleles of pre-(A) and post-fragmentation (B) cohorts, as a function of sample size. The standard deviation obtained by randomly generating 10000 replicates of alleles for every sample size. This permutation procedure was used to assess the robustness of number of alleles to unevenness in sample size. Solid and dashed lines are mean values and confidence intervals from standard deviation, respectively. Red, black and green lines are those of slight-, moderate-and strong-fragmentation types, respectively.

At the cohort level, a significant relationship was also observed between the number of alleles per locus and habitat size for both pre-and post-fragmentation cohorts, and there was a significant difference between the two regression curves (*F* = 5.23, P = 0.014) (Fig. 4). Post-fragmentation cohorts had lower numbers of alleles per locus in small habitats, while similar values in pre-and post-fragmentation cohorts occurred in large habitats (Fig. 4). A re-sampling procedure demonstrated the relatively low number of alleles is robust to unevenness in sampling size: both pre-and post-fragmentation cohorts of each habitat type displayed similar genetic diversity (Fig. 5). Similar Fisher's α index was found in the three habitat types, and Fisher's α indices of pre-fragmentation cohorts (6.55, 6.18 and 6.09 for slightly, moderately, and strongly fragmented habitats, respectively) were lower than those of post-fragmentation cohorts (7.95, 6.58 and 8.94 for slightly, moderately and strongly fragmented habitats respectively), also indicated fragmentation per se has not negatively affected genetic diversity of *C. sclerophylla* populations.

### Genetic diversity at chloroplast microsatellite loci

Three of the six cpDNA microsatellite loci were polymorphic with a total of 10 alleles. Combining information from those alleles allowed the identification of five haplotypes among the 460 individuals. Haplotype II was the most frequent overall while haplotypes IV and V were found in only one individual, in populations XS and LS, respectively. Eight populations contained only one haplotype, while population XS had three haplotypes. The mean Nei's gene diversity of the populations was 0.010, and the Nei's gene diversity of the global population was 0.102. In the studied region, chloroplast haplotypes exhibited a distribution gradient (Fig. 1), indicating limited seed dispersal. Haplotype I dominated six populations located in the eastern part of the study area, while haplotype II dominated seven populations located in the central and northern parts. Haplotype III was found in all individuals of population AC, located in the extreme west, and in one individual of population WM, located in the eastern part of the study area.

The number of haplotypes was marginally significantly related to habitat size (adjusted *R* ^2^ = 0.1524, P = 0.092). Based on this relationship, the predicted number of haplotypes of an imaginary population of a size equaling the total area of the islands was 1.91, much smaller than the observed value (3 haplotypes) in the island populations.

### Pollen-vs. seed-mediated gene flow

The estimated
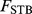
was 0.066, and was significantly larger than zero by bootstrapping over loci. Based on chloroplast haplotypes, we found a large differentiation among populations (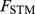 = 0.893). The calculated inbreeding coefficient (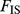) was 0.390. Under the assumption of an island model, levels of differentiation at biparentally inherited and maternally inherited markers indicated gene dispersal by pollen was much more extensive than by seed among populations of *C. sclerophylla* in the study area (*r* = 162.2). Post-fragmentation cohorts had a higher *F*_IS_ than pre-fragmentation cohorts (0.380 v.s. 0.348), and a higher *F*_ST_ by cpDNA SSRs (0.915 v.s. 0.878). However, nuclear *F*_ST_ values were nearly the same in pre-and post-fragmentation cohorts (0.073 v.s. 0.072). The calculated values of pollen/seed dispersal ratios were 121.2 and 189.5 in the pre-and post-fragmentation cohorts, respectively.

## Discussion

### Effects of habitat loss and fragmentation per se on nDNA genetic diversity

Habitat destruction leads to habitat loss and fragmentation per se, resulting in a decrease in genetic diversity. Fifty years after dam construction, we found a positive relationship between habitat size and number of alleles per locus using nuclear microsatellites or number of cpDNA haplotypes, a pattern indicating smaller habitats harbor populations of lower genetic diversity. This pattern was frequently observed in the fragmented populations [2], [3]. Using genetic diversity-habitat size relationships, we found the number of alleles per locus and the number of haplotypes of island populations were higher than those of an imaginary population of size equaling the total area of the islands, hinting that a single large habitat may contain lower genetic diversity than several small habitats with the same area. A re-sampling procedure demonstrated relatively low genetic diversity of populations in small habitats is robust to unevenness in sampling size. These results indicate habitat loss other than fragmentation per se played a major role in the decline in genetic diversity in remnant *C*. *sclerophylla* populations in the studied region.

It is not surprising that habitat loss leads to reduced genetic diversity of both nuclear and chloroplast genomes of *C. sclerophylla* populations. Habitat loss directly decreases the area of suitable habitats, resulting in a decrease in population size or even leading to local extirpation of some species. Genetic diversity may also decline during this process. The positive relationship between both habitat area or population size and genetic diversity observed in many studies confirmed the negative impact of habitat loss [41]. Such negative impacts are thought to play a major role in the decline of biodiversity during the process of fragmentation [1].

That fragmentation per se had not played a major role in genetic diversity in the fragmented landscape seems to contrast with most previous observations [2], [3]. There might have no enough time for genetic drift and inbreeding to play a substantial role in the present study. Previously reported distinct impacts of fragmentation on genetic diversity were mostly found in short-lived plant species [2], [3]. In trees, only few cases have reported significant changes in genetic variation caused by fragmentation [18], [42]. For example, in fragments at least 600 years old, Jump and Penuelas [42] detected negative genetic impacts, i.e., elevated levels of inbreeding and divergence and reduced genetic diversity, caused by forest fragmentation in a widespread wind-pollinated *Fagus sylvatica* L. In the present study, *C. sclerophylla* populations have been fragmented for only five decades, i.e., less than two generations. Also, *C. sclerophylla* may resprout, which permits persistence in demography and prolongs generation time [43], and thus reduces the negative impacts of genetic drift and inbreeding in fragmented habitats.

Another plausible reason is that fragmentation per se has weak or no negative or even positive impacts on genetic diversity, as observed in species diversity [37]. Previous studies on the genetic consequences of fragmentation did not separate the effect of fragmentation per se from the effect of habitat loss. After decoupling the two effects, Yaacobi *et al.* [37] found fragmentation per se had not influenced the number of species present in a highly fragmented Mediterranean scrub landscape. Some previous theoretical and empirical studies also suggested that subdivision of the same amount of habitats into many smaller pieces can have positive effects on biodiversity [1], [44]. Honnay *et al*. [45] also found historical and present landscape configuration had a low impact on genetic variation in fragmented populations of *Anthyllis vulneraria*, a rosette-forming legume. Our study shows a large population contains lower genetic diversity than several small ones, indicating that fragmentation per se may have a positive impact on genetic diversity in *C*. *sclerophylla*. It is well known a heterozygous landscape may maintain high genetic diversity caused by differential selection [46]. However, this may not hold in the present study because microsatellites are generally neutral. A possible reason is that several small populations may cover a much larger and more isolated area than a large one covers. Given limits to dispersal, fragmented populations may have a high possibility of containing more genetic diversity than unfragmented populations. This was evidenced by the difference in genetic diversity mediated by pollen and seed (Fig. 2).

### Effects of habitat loss and fragmentation per se on cpDNA genetic diversity

Compared to nuclear DNA diversity, chloroplast DNA genetic diversity in *C. sclerophylla* populations seems to be more tolerant to fragmentation, which has also been observed in other plant species, such as oaks in France [14] and *Pinus elliottii* var. *densa* [15]. Petit *et al*. [14] suggested comparable cpDNA diversity of oaks in fragmented habitats was caused by increased seed flow which was caused by fragmentation or by human activities. However, in *C. sclerophylla*, low cpDNA diversity within populations may have limited the effects of fragmentation per se. When seed dispersal is limited, most populations had only one haplotype. Although some other populations had two or three haplotypes, the frequency of the dominant haplotype was lower than 0.9 in only two populations (Table 1). Also, *C. sclerophylla* has a strong ability to resprout. When disturbed, *C. sclerophylla* regenerates mainly by resprouting [11]. Therefore, isolation caused by habitat destruction may have no substantial impact in the short-term on cpDNA diversity. At an extreme, some studies failed to find any polymorphism in cpDNA, and all populations, no matter large or small, have the same haplotype [47]–[49].

Since *C. sclerophylla* is wind-pollinated and gravity-dispersed, it has a high pollen-to-seed dispersal ratio. This ratio is among the highest for plants at a range-wide scale, and much higher than 17, the median ratio of 93 taxa [12]. Most species of Fagaceae have high pollen-to-seed dispersal ratios caused by the combination of wind-pollination and gravity-dispersal. Pollen-to-seed dispersal ratios of Fagaceae species were larger than 200 [50]. Compared to these values, the pollen-to-seed dispersal ratio in *C. sclerophylla* was small because of the limited range in the present study, as scale plays a critical role in estimating the ratios [51]. At a local scale, pollen-to-seed ratios may be much smaller than at a regional scale. For example, across a range of about 2 km^2^, pollen-to-seed dispersal ratio was 1.15–2.16 in the congener *C. fargesii* Franch. [52], indicating that seed dispersal may play a comparable role in pollen dispersal at a fine scale.

In conclusion, by decoupling the effects of habitat loss and fragmentation per se, we found, in recently fragmented habitats, habitat loss may play a major role in the decrease of nuclear and chloroplast genetic diversity of *C. sclerophylla*, while fragmentation per se has no negative effect, but does have a weak positive impact. Our results suggest habitat loss may explain the early decrease in genetic diversity, while fragmentation per se may play a major role in differentiation among fragmented populations and a later decrease in genetic diversity.
